# Prejudice and Feeling of Threat towards Syrian Refugees: The Moderating Effects of Precarious Employment and Perceived Low Outgroup Morality

**DOI:** 10.3390/ijerph17176411

**Published:** 2020-09-03

**Authors:** Macarena Vallejo-Martín, Jesús M. Canto, Jesús E. San Martín García, Fabiola Perles Novas

**Affiliations:** Department of Social Psychology, Social Work, Social Anthropology and East Asian Studies, Faculty of Psychology, University of Malaga, 29071 Málaga, Spain; jcanto@uma.es (J.M.C.); sangar@uma.es (J.E.S.M.G.); fanovas@uma.es (F.P.N.)

**Keywords:** prejudice, refugees, realistic and symbolic threat, outgroup morality, precarious employment

## Abstract

Refugees frequently experience traumatic situations that result in the deterioration of their psychological well-being. In addition, perceived prejudice and discrimination against them by the host society can worsen their mental health. In this research study, using a Spanish sample, prejudice towards Syrian refugees is analyzed taking into account feeling of threat (realistic or symbolic), precarious employment, and perceived outgroup morality. Using a total of 365 participants, our results reveal that individuals feel more prejudice towards refugees when the former scored higher in realistic threat and symbolic threat, were in a highly precarious situation of employment and perceived refugees as being more immoral. Furthermore, it was found that persons who scored high in realistic threat and at the same time were in a situation of precarious employment, were those who displayed greater prejudice. The results likewise pointed to individuals who scored high in symbolic threat and in outgroup morality as being those who felt greater rejection towards the refugees. Accordingly, our results confirm the importance of feeling of threat in relation to prejudice, and highlight two important moderating factors: precarious employment and perceived outgroup morality.

## 1. Introduction

According to the United Nations High Commissioner for Refugees (UNHCR) [1], in recent years we have been immersed in a humanitarian crisis which has led to the displacement of millions of people due to wars or violence in their countries of origin. Specifically, it is estimated that there are 70.8 million forcibly displaced people in all of the world, with 25.9 million of them being refugees. This phenomenon called “the refugee crisis”, is characterized by the growing number of forced displacements toward Europe and other Western countries [2]. The main countries of origin are Syria, Afghanistan, South Sudan and Myanmar [1]. In fact, the case of Syria represents the largest exodus that has taken place since World War II [3].

The circumstances causing the refugees to flee their countries make this displacement different from other types of voluntary migration, as it is frequently characterized by forced departure, fear and traumatic experiences. This feature often leads to refugees experiencing, once they are settled, mental health problems and a significant deterioration in their mental health [4,5] and wellbeing [6]. In particular, refugees have a high prevalence of mental disorders, specifically post-traumatic stress disorder and depression [7,8]. Research carried out by Chung et al. [9] on Syrian refugees shows that 43% of them have post-traumatic stress disorder and that they have higher levels of psychological stress when they have witnessed situations of horror, death, etc. In addition, the study highlights differences in the mental health of refugees depending on the host country. On the other hand, the degree of acceptance and integration of the host country is also a key element for the mental health of this sector of the population, since there is a negative relationship between perceived prejudice and the psychological wellbeing of potentially stigmatized minority groups [10]. In this sense, the meta-analysis undertaken by Pascoe and Smart Richman [11], which takes into account the results of 134 studies carried out in diverse countries, revealed that stress levels increase when there is perceived discrimination. Until now there have been few studies that analyze which factors contribute to prejudice and discrimination of the host country towards refugees. 

At the same time, the impact of perceived threat of ethnic prejudice is well documented [12]. Nevertheless, until now there have been no studies that have taken an analysis of outgroup threat in relation to prejudice towards refugees into account in the Spanish context, with this being an essential variable for the understanding of intergroup relations, formation of negative stereotypes towards outgroups [13,14,15] and as such, for mental health and psychological wellbeing.

### 1.1. The Importance of Outgroup Threat in Understanding Prejudice

Cottrell and Neuberg [16] hold that the specific emotions that we feel toward other groups arise from the perception that such groups threaten certain aspects that are important to us, such as, for example, economic resources or values. Accordingly, when individuals perceive that members of other groups put into question or threaten elements they value, they experience outgroup hostility. In recent years, the Intergroup Threat Theory (ITT) [17] has become an important theoretical framework for understanding the key role of threatening elements in the genesis of ethnic prejudice, taking into account one of the most relevant approaches in this field [18]. The ITT distinguishes between two basic sources for threat: realistic threat and symbolic threat. Realistic threat implies the perception of competition between ingroup and outgroup for scarce resources, such as employment, social services, education and healthcare [19]. At the same time, symbolic threat refers to the perception of differences in values and beliefs that members of outgroups are thought to have. In this sense, symbolic threat is focused on worldview and moral values, along with fear of losing ingroup customs, language and traditions as a consequence of interaction with members of the outgroup [20].

The ITT, and its previous versions [21,22], have demonstrated that perceived threat, both symbolic and realistic, plays a central role in intergroup attitudes, and it is a predictive variable for prejudice in various social contexts [17]. This relation between intergroup threat and greater resentment and hostility towards immigrants has been reflected in different studies, for recently arrived immigrants [23,24] as well as for those who are settled residents [25]. However, different research studies have brought to light that the degree to which each type of threat is related to prejudice depends to a large extent on the nature of the relation between the groups being considered [26]. Stated in another way, different minority groups can provoke different perceptions of threat [17,27,28]. Due to the fact that the type and importance of the threat induced depend on the specific characteristics of the intergroup context, different outgroups can evoke differential attitudinal reactions among members of the majority group [29]. Along these lines, one of the most recent studies is that of Jedinger and Eisentraut [18]. These authors hypothesize an effect of different types of threat on ethnic prejudices based on perceived characteristics in minority groups. The results of this study, undertaken within the German context, show that negative attitudes towards Muslims and third-generation Turks arise mainly from a perception of economic threat and cultural threat, while the refugees and the Romani are also linked to threat involving public safety and criminality. In the Spanish context, the relation between hostility towards immigrants and perceived realistic and symbolic threat have been dealt with in different studies [30,31,32,33]; nevertheless, until now, this relationship has not been studied in the refugee population.

### 1.2. Stereotype Content and the Dimension of Outgroup Morality 

Although in colloquial terms concepts such as prejudice, discrimination, racism, and stereotypes are used interchangeably, we should bear in mind that they are not synonymous concepts. Traditionally, prejudice has been conceived as a negative attitude towards a certain social group [34], but nowadays the role of emotions has become especially relevant. From the theory of the inter-group emotions [35], prejudice is considered as an emotion that depends on the social, political, and cultural context in which intergroup relations take place [36]. On the other hand, social stereotypes are beliefs about characteristics that a group of people have stemming from simply belonging to a social group [37]. The question as to why we associate different attributes to certain social groups has been a recurrent one in the study of stereotypes. In general, it has been confirmed that the formation of stereotypes is a complex phenomenon that can be explained by cognitive, affective, socio-motivational, and cultural processes [38]. 

According to the Stereotype Content Model [39,40], judgement about others derives from structural relationships that the groups maintain with one another. These relationships are determined by whether these groups compete for resources or if they are high or low status. For the model’s first approaches [39,40], the social perception towards the outgroup is developed on the basis of two dimensions: warmth and competence. Later, Leach, Ellemers and Barreto [41] established that the dimension of warmth in fact encompasses two different dimensions: sociability and morality. Sociability is associated with the desire to interact with others (for example, being friendly or nice). At the same time, morality refers to the degree to which the behavior of the person or group evaluated is considered proper (for example, a trustworthy or sincere person). The existence of these two differential dimensions and their impact on prejudice has been confirmed in diverse studies [42,43,44,45]. Furthermore, it seems that the dimension of morality is the one with most weight when it comes to determining our evaluation of certain groups [46,47].

One of the most recent studies on refugees in the Spanish context corresponds to that by Ordóñez-Carrasco, Blanc, Navas and Rojas-Tejada [48]. In this research, the dimension of outgroup morality is linked [42] to the acculturated preferences towards Syrian refugees. According to these authors, when the host population perceives a high degree of outgroup morality, and as such, the outgroup is no longer a threat, the former are seen as more flexible and permissive with regard to the outgroup maintaining its culture of origin in the private sphere. Our study, thus, points out the importance of perceived morality in prejudice and in the strategies of acculturation, not only with respect to immigrants, but also individuals who are refugees. 

### 1.3. The Present Study

In this study, we seek to determine the role of outgroup threat in its two dimensions, realistic and symbolic, in the ethnic prejudice of the Spanish population towards Syrian refugees. In Europe, two types of opposing discourse converge with respect to refugees [49]. On one hand, there is the discourse where human rights take precedence and there is a call for countries to offer protection to people coming from other ones that are in conflict; on the other hand, there is other discourse that highlights the risk and threat represented by mass acceptance of people who have been forced to emigrate [48]. In recent years, in Spain, the number of people requesting asylum has grown exponentially, going from 5947 in 2014 to 118,264 in 2019 [50]. Specifically, according to Spanish Commission for Refugees (CEAR) [50], the number of Syrian refugees in Spain is estimated to be between 12,000 and 15,000 people. The latest data on Syrian asylum seekers in Spain, corresponding to 2019, indicated a number of 2775. This increase within such a short time is important in the host population’s perception of refugees, because among the main variables involved in the activation of threat and negative attitudes towards outgroups are the size of the foreign population and competition for resources brought about by the country’s economic context [22,31,51]. In line with the ITT [17], in our study we expected the participants to show greater prejudice when they felt a higher degree of realistic threat (H1a) and symbolic threat (H1b) toward Syrian refugees. 

At the same time, the refugee crisis is framed within the context of the economic and financial crisis in Europe, which, together with the austerity measures, have brought about a lesser degree of openness from the European population towards persons coming from other countries [52]. During periods of crises, the perception that minority groups pose a threat for scarce resources and for the national cultural homogeneity becomes exacerbated [51]. Specifically in Spain, the economic crisis of 2008 was severe and intense and intergroup relations were sharply impacted by this social reality. This socioeconomic context diminished the quality and level of life of Spain’s citizens (Spaniards and immigrants), who have had to compete for jobs and social resources in a society in crisis for a decade [30]. In this sense, we expect to obtain that those persons that were in a situation of precarious employment, defined in this study as individuals who are unemployed or employed in a job that they viewed as having very negative conditions, would show greater levels of prejudice towards refugees (H2). In addition, as realistic threat implies perceived competition for resources, among which is employment [22], we expected that the situation of precarious employment would be a moderating factor for the same. Accordingly, we hypothesized that individuals would feel greater hostility towards Syrian individuals when they scored high in realistic threat and were in situation of precarious employment (H3).

Finally, the dimension outgroup morality was taken into account in the study, which, as stated earlier, is considered the most important for the stereotype content and one of the most relevant factors in outgroup hostility [42,53]. In line with results from other studies on ethnic prejudice towards minority groups in the Spanish context [43,48,54], it was expected that when individuals perceived low morality in the Syrian refugees, they would show greater prejudice towards them (H4). Furthermore, we hypothesized that perceived morality would be a moderating effect for symbolic threat. Thus, it was expected that when individuals perceived that their main traditions and values were endangered [20] by the arrival of Syrian refugees, that is—they felt threatened by them in the symbolic dimension, and at the same time, felt that they were immoral [42], harmful and dishonest persons (H5)—they would show greater outgroup hostility.

## 2. Materials and Methods 

### 2.1. Participants

A total of 472 individuals participated in this study, from which those who were not in a position to be employed (such as students, retirees, people with disabilities, etc.) were discarded, resulting in a final total of 365. Among the participants, 51.5% were women and 48.5% men. The average age was 40.35 (*SD* = 11.61), in ages ranging between 19 and 67. As for education level, the distribution was the following: 21.9% basic education, 28.1% secondary education 28.8% university studies, and 21.3% vocational/professional training. Regarding employment status, 17.6% were unemployed and 28.5% were employed and viewed their job in a negative light, while 53.9% had a job which they evaluated positively and as being in line with their expectations.

### 2.2. Instruments

Sociodemographic questionnaire. The participants were asked about sociodemographic characteristics such as their sex, age, educational level, employment status, and political viewpoint. 

Outgroup Threat Perception Scale. To measure outgroup threat, the Escala de Percepcion de Amenaza Exogrupal-EPAE (Outgroup Threat Perception Scale) [32] was used. It is composed of a total of 13 items which range from response 1 (“Not at all”) to 5 (“Very much so”) that assess two factors: realistic threat (9 items) and symbolic threat (4 items). The items to measure symbolic threat refer to the degree to which the individuals feel that refuges endanger educational and family values, religious beliefs and cultural traditions. At the same time, the items that assess realistic threat indicate the extent to which individuals feel that the refugees put at risk access to jobs, healthcare, education, welfare benefits, the economic stability of the country, health, public order, and personal and national security. The Cronbach’s alpha obtained was 0.87 for symbolic threat and 0.89 for realistic threat.

Stereotyped dimension of outgroup morality. A scale was designed based on the research of Leach, Ellemers and Barreto [41] in the Spanish version [43]. Participants were asked to what degree they considered the refugees honest, trustworthy, sincere, respectful, fair and well-intentioned for a range of responses that went from 1 (“Not at all”) a 5 (“Very much so”). The reliability coefficient was 89.

Prejudice. A scale of emotions was used [55], composed of 10 items with responses ranging from 1 (“Not at all”) to 5 (“Very much so”), which measured the affective component of the prejudicial attitude towards refugees through negative emotions (fear, unease, anger, disgust, hate, deception, disdain, frustration, resentment, and agitation). The Cronbach’s alpha obtained was 0.92.

### 2.3. Procedure

The data were collected in Malaga (Spain). The questionnaire was administered by doctoral students from the Faculty of Psychology and Speech Therapy at the University of Malaga, who had been previously trained in social and community research. The sample was random using the snow-ball sampling method [56], asking for voluntary collaboration from participants. The questionnaire was anonymous and data confidentiality was assured as was the fact that results would only be used for academic purposes. In addition, participants read a debriefing explaining the goals of the study and they also were able to request an additional oral debriefing. The ethical guidelines of this research were approved by the Research Ethics Committee of the University of Málaga (85-2019-M). The data were gathered between November 2019 and February 2020.

### 2.4. Statistical Analysis

Once the data screening was performed, the SPSS v23.0 statistical software (SPSS Inc., Chicago, IL, USA) was used to calculate the reliability, descriptive statistics, and correlations of the measured variables. First, the reliability was calculated for each of the scales used for the variables realistic threat, symbolic threat, outgroup morality and prejudice. With respect to employment status, participants were classified into two groups: (1) highly precarious employment: this includes unemployed individuals or individuals who perceive their job as having very negative work conditions and (2) low degree of precariousness: composed of individuals who have a job that they view as being in accordance with their expectations and with good working conditions. Later, the descriptive statistics were measured (mean and standard deviation) and the correlation among variables. Lastly, a hierarchical regression analysis was carried out through the PROCESS macro for SPSS [57].

## 3. Results

First, the descriptive statistics and the correlations between variables were calculated. The results show that the participants obtained a medium-low score in realistic threat (*M* = 2.22; *SD* = 1.10) and symbolic threat (*M* = 2.11; *SD* = 1.07), medium-high in outgroup morality (*M* = 3.17; *SD* = 0.89) and low in prejudice towards the refugees (*M* = 1.49; *SD* = 0.91). As for the relationship between the variables, it was observed that for higher levels of realistic threat, there were higher levels of symbolic threat (*r* = 0.597; *p* < 0.01), higher levels of prejudice towards refugees (*r* = 0.381; *p* < 0.01), and lower levels of perceived morality (*r* = −0.450; *p* < 0.01). At the same time, at higher levels of symbolic threat there were greater level of prejudice towards refugees (*r* = 0.401; *p* < 0.01) and lower levels of perceived morality (*r* = −0.499; *p* < 0.01). It was also found that with higher levels of outgroup morality there was lower prejudice (*r* = −0.422; *p* < 0.01). 

Then, a hierarchical regression analysis was carried out (Table 1), in which the criterion variable prejudice towards refugees (R^2^c = 0.399; *F_(4, 365)_* = 21.938, *p* = 0.001). In the first step, age and gender included as control variables, not being statistically significant (gender: *β* = 0.017, *t* = 0.068, *p* = 0.946; age: *β* = 0.190, *t* = 0.792, *p* = 0.440). In the second step, four predictor variables were included: realistic threat, symbolic threat, outgroup morality and precarious employment. Finally, in the third step, the interactions between realistic threat and precarious employment and symbolic threat and outgroup morality were included. All of these variables had a significant effect on greater prejudice towards refugees. 

**Figure 1:**
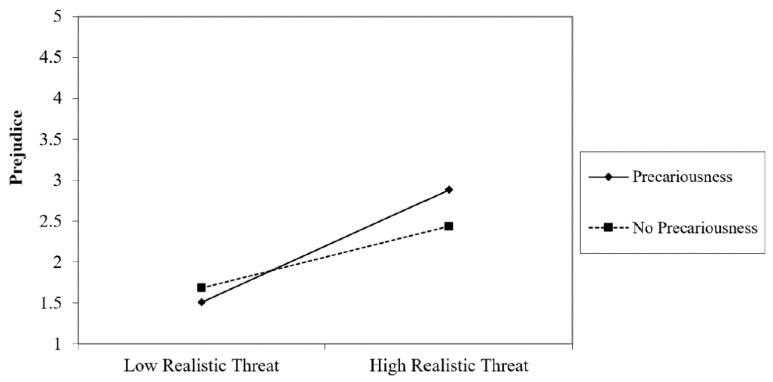
Interaction between Realistic Threat and Precariousness on Prejudice.

**Figure 2:**
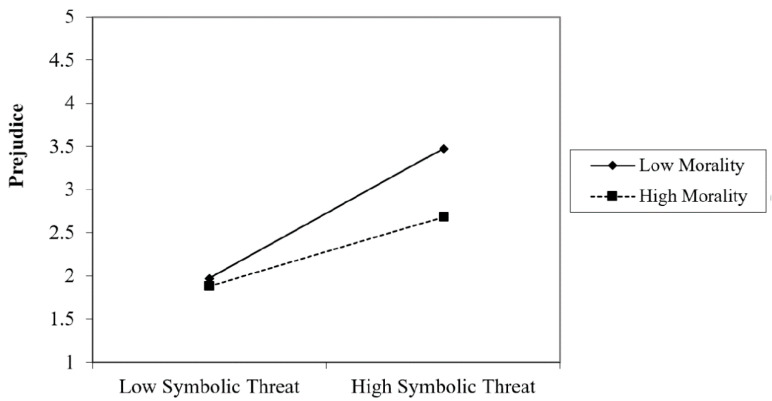
Interaction between Symbolic Threat and Morality on Prejudice.

Individuals showed more prejudice when they felt threatened by the refugees in the realistic dimension (*β* = 0.447, *t* = 2.07, *p* = 0.020) and the symbolic one (*β* = 0.759, *t* = 3.53, *p* = 0.001), perceived low morality in the refugees (*β* = −0.276, *t* = −3.70, *p* = 0.000) and were in highly precarious employment (*β* = 0.146, *t* = 2.33, *p* = 0.021). It was likewise found that the interaction between realistic threat and precarious employment was significant (*β* = 0.561, *t* = 2.65, *p* = 0.009). To understand the interaction between variables we follow the process suggested by Aiken and West [58]. The test of simple slopes revealed that the realistic threat of participants predicted prejudice when they were in highly precarious employment ((+1 SD) (*β* = 0.439, *t* = 5.55, *p* = 0.000) but not when precarious employment was low (−1 SD) (*β* = 0.137, *t* = 2.09, *p* = 0.057). That is, individuals showed more prejudice when they perceived a higher threat regarding resources with respect to refugees and were in a situation of precarious employment. (Figure 1). At the same time, interaction between symbolic threat and outgroup morality was significant *(β* = −0.300, *t* = −4.40, *p* = 0.000). The test of simple slopes revealed that symbolic threat in the participants predicted prejudice when they showed low levels of outgroup morality (−1 SD) (*β* = 0.260, *t* = 3.91, *p* = 0.000), but not with high levels (+1 SD) *(β* = 0.012, *t* = 1.40, *p* = 0.888). That is, when individuals perceived a risk to their values and customs from the Syrian refugees and thought that the latter were not honest or trustworthy, more prejudice was felt (Figure 2).

## 4. Discussion

In this study we have sought to study the relationship of the feeling of threat, level of employment precariousness, and perception of outgroup morality with prejudice towards Syrian refugees in the Spanish context. The majority of refugees experience diverse emotional problems and a deterioration in their psychological wellbeing as a consequence of the traumatic experiences they live through [59]. The response of the host society during their arrival and the perception of being accepted can be key to their integration and recovery [60] and, in contrast, perceived discrimination can generate disorders such as depression and anxiety [61,62].

In keeping with the ITT [17], outgroup threat plays an important role in understanding outgroup hostility towards the collective studied. In this sense, our results are in line with what was posited in H1: the host population shows more prejudice when it feels threatened by the Syrian individuals, due to competition for resources (H1a) and because the refugees could put their values, traditions and worldview at risk. (H1b). Different studies along these lines have corroborated the relationship between hostility towards minority groups and realistic threat [12,13,17,63] as well as symbolic threat [25,64].

On the other hand, our H2 postulated that individuals who were in a situation of highly precarious employment would experience more negative emotions towards Syrian refugees. Indeed, our results are in line with what we expected, with individuals in highly insecure employment being those who showed greater outgroup hostility. From the classic theory of realistic threat for analyzing prejudice [65], it was established that when groups compete for resources, more intense intergroup rejection behavior and emotions arise, leading to increased prejudice and discrimination. In addition, according to our data, the situation of precarious employment is a moderating factor in the feeling of realistic threat, as was formulated in H3. When individuals scored high in this dimension of threat and are in a situation of precarious employment more prejudice towards Syrian refugees was displayed. This relationship is worthy of note, given the current healthcare crisis, which is also provoking a social and economic crisis, considerably increasing levels of unemployment. Specifically, in Spain following the outbreak of the COVID-19 pandemic, the current unemployment rate (May 2020) is at 14.4%. In situations of crisis, the perception of threat increases [51] and minority groups (for example, immigrants and refugees) can become scapegoats for taking out frustration [66] and consequently prejudice is increased. Therefore, fostering policies of employability and improving work conditions not only foment a more socially cohesive society, but also one where potential intergroup conflicts can be prevented. 

Lastly, in this study we have taken into account the stereotyped dimension of morality, so that when individuals perceive the Syrian refugees as not being honest or trustworthy, they showed more prejudice, in accordance with H4. Different studies [46,47] have determined that the dimension of morality is the one carrying most weight when determining valuations of certain groups and is a determining factor in regards to prejudice [42,53]. Furthermore, in our study we have been able to verify that morality is a moderating factor for symbolic threat, as was posited in H5. When individuals feel that Syrian refugees constitute a threat to their worldview, traditions and ways of understanding life and presuppose that these have unethical or socially harmful values, they display a higher degree of outgroup hostility. 

### Limitations and Future Research

As a main limitation of our study, we point out that in our research the consideration of individuals with a highly precarious employment has been made up of two elements: an objective one, being unemployed, and subjective, one’s own assessment of their work conditions as being negative, understanding that the feeling of threat from outgroups can be activated in the lowest socio-economic groups and among those with fewer resources. In future studies, it would be worthwhile verifying if there are differences with respect to the level of prejudice and the perception of threat by individuals who are unemployed contrasted with those who, although are working, consider their employment to be of low quality. At the same time, it should be analyzed, if after the COVID-19 pandemic, with the resulting higher unemployment rate (which is currently about 20%), the negative conception and rejection of refugees has increased. It would also be important to know if there are differences in the level of prejudice according to the level of education and the perceived social class.

Along these lines, it would be of importance to check for possible differences in the stereotypes towards different groups of refugees, for example, towards Venezuelans, who represent the largest group of refugees in Spain [50]. We also consider it relevant to study the possible differences that may exist in psychological well-being between the local population and refugees. Likewise, it would be convenient to analyze the perception of rejection felt by the refugee population since the threat of stereotyping in minority groups can increase anxiety and deteriorate mental health [67].

Lastly, we should add that, in future research, it would be of value to consider ideological viewpoints in relation to prejudice. In this vein, the study carried out in different European countries by Wike, Stokes and Simmons [68] points out that individuals with a right-wing political ideology are more concerned about the arrival of refugees and show more negative attitudes towards minorities (especially Muslims). At the same time, the ideological variables of right-wing authoritarianism [69] and social dominance [70] have been widely related to prejudice [71,72,73] and outgroup threat [74].

## 5. Conclusions

According to the Geneva Convention [75], states must share responsibility for taking in those individuals who have to flee their countries of origin because they are in danger. However, European countries, until now, have not developed a true policy of integration for this collective. This inevitably leads to the host population feeling threatened and to the existence of different elements that can increase prejudice. In the shared beliefs held regarding outgroups, cultural factors, socialization processes and political, social and economic circumstances all play a central role [76]. The degree of understanding and framework in which intergroup relations are established have an effect at the collective level, according to the group they are immersed in, as well at the individual level. In the case of the individuals making up the minority groups, the perception of societal rejection and discrimination could lead to a loss of psychological wellbeing, and as such, deterioration in mental health [61]. The fact that at this moment, Syria is one of the countries with the highest number of displaced persons as a consequence of the war [1], and that a significant volume of refugees have arrived in Spain within a short span of time [50], makes it necessary to attend to this social reality for the sake of constructing a more cohesive society, fomenting equality among fellow citizens and improving the mental health of the refugee collective. 

In this study, we have been able to confirm that the feeling of threat towards refugees is moderated by the perception of employment precariousness and outgroup morality—elements that must be taken into consideration for improving the inclusion and integration of this collective and for developing positive intergroup relations. The results of this research show that for future intervention programs aimed at reducing racism and xenophobia, it is important to deconstruct the perception of refugees as a threat, to seek empathy with this group and to highlight their possible contribution to the host society.

## Figures and Tables

**Table 1:** Multiple regression analysis on prejudice.

		*β*	*t*	*p*
Step 1				
	Gender	0.017	0.068	0.946
	Age	0.190	0.792	0.440
	R^2^c = 0.014			
Step 2				
	Realistic Threat	0.447	2.07	0.040
	Symbolic Threat	0.759	3.53	0.001
	Outgroup Morality	−0.276	−3.70	0.000
	Precariousness	0.146	2.33	0.021
	ΔR^2^c = 0.301			
Step 3				
	Realistic Threat × Precariousness Symbolic Threat × Morality	0.561 −0.300	2.65 −4.40	0.009 0.000
	ΔR^2^c = 0.084			
